# A cross-sectional study of surgical glove perforation during the posterior lumbar interbody spinal fusion surgery: Its frequency, location, and risk factors

**DOI:** 10.1097/MD.0000000000010895

**Published:** 2018-06-01

**Authors:** Min Seok Kang, Yeong Ryung Lee, Jin Ho Hwang, Eun Taek Jeong, In Seok Son, Suk Ha Lee, Tae Hoon Kim

**Affiliations:** a Department of Orthopedic Surgery, Seoul Red Cross Hospital; b Department of Orthopedic Surgery, CM General Hospital; c Department of Orthopedic Surgery, Konkuk University Medical Center, Konkuk University School of Medicine, Seoul, Korea.

**Keywords:** bone trimmer, glove perforation, posterior lumbar interbody fusion, risk factor, surgeon

## Abstract

Lumbar fusion surgery was known to pose a greater risk of surgical glove perforation. However, there has been no study on the glove perforation that can transmit the blood-borne disease to the patient and surgical staff members in the posterior lumbar interbody fusion surgery (PLIFs).

We performed a cross-sectional study to investigate the glove perforation during the PLIFs. The study included 37 consecutive patients (10 males and 27 females). All used gloves of surgical staff members, which included the surgeon, assistant surgeons, bone trimmer (who performed local bone trimming and interbody cage preparation), and scrub nurse were collected and were performed to the pinhole water infusion test. The characteristics (i.e., frequency and location of perforated glove) and relative risk of glove perforation were investigated for each participant. The independent risk factors influencing glove perforation were analyzed by multiple logistic regression analysis.

The overall operative perforation rate which is a percentage of detected more than one glove perforated event in all cases was 51.4%. The overall glove perforation rate which is the percentage of perforated gloves in all gloves used for surgery was 3.8%. The relative risk of glove perforation by each participant was 2.38 in the surgeon (*P = *.002), 1.36 in the bone trimmer (*P = *.04), 1.36 in the scrub nurse (*P = *.04), and 1.19 in assistant surgeons (*P = *.13). And, the volume of trimmed local bone was analyzed as an independent risk factor for glove perforation (ORs = 1.310, *P = *.02).

The overall operative perforation rate in PLIFs is higher than 50%. The surgeon, scrub nurse and bone trimmer were observed as a significant risk factor for glove perforation. And, the volume of trimmed local bone was analyzed as independent risk factor. Since the preparation of the interbody cage is essential for successful lumbar fusion surgery, the bone trimmer must pay attention to the glove perforation during this procedure.

## 1 Introduction

Some studies have shown that glove perforation is more common in orthopedic surgery than in any other surgical procedures.^[1–5]^ A cause of this high occurrence includes dealing with sharp bony fragments and the use of needles and sharp and complex surgical instruments. Lumbar interbody fusion surgery poses particularly high risk of perforation due to multiple manipulations with surgical instruments and the presence of sharp bones during local bone graft preparation.^[6]^

Surgical gloving is important not only to prevent infection of patient, but also to protect surgical staff against blood-borne diseases, such as HIV, HBV, and HCV infections.^[7]^ Unnoticed Surgical glove perforations during operation is a recognized pathway through which surgeons and other members of operation staffs could be exposed to patient's blood.^[5]^

Identification of factors that cause glove perforation in posterior lumbar interbody spinal fusion surgery (PLIFs) is important for finding a better solution to the problem. The purpose of the current study was to assess the frequencies, specificities and contributing factors of surgical glove perforations in PLIFs.

## 2 Materials and methods

### 2.1 Study subjects

This study was approved by the Institutional Review Board of our institute (KUH1060154), and all patients provided informed consent. The surgical glove perforation rate was prospectively measured during surgery performed on 37 consecutive patients (10 males and 27 females) who underwent PLIFs for lumbar degenerative disease from April 2015 to February 2016. Inclusion criteria were adult patients between 20 and 80 years of age, with degenerative lumbar spinal pathology. Patients were excluded if they had tumor, infection, trauma, or previous lumbar surgery). All surgeries were conducted by the same surgical team. All surgical staff, which including the surgeon, first and second assistant surgeons, bone trimmer (who performed local bone trimming and cage manipulation), and scrub nurse wore conventional double surgical gloves (Triflex custom sterile latex surgical gloves, Cardinal Health, Waukegan, IL). All surgical staff members who participated in the surgery were right handed. Also, all clinical data were obtained included age, gender, surgical running time, level of fusion segment, volume of estimated blood loss, and volume of trimmed local bone.

### 2.2 Operative technique and assessment of glove perforation

Under general anesthesia, the patient was placed on the operating table in a prone position on a lumbar frame. Draping was performed while wearing inner gloves. All surgical staff members put on the outer gloves just before surgery. Using a median posterior approach, pedicle screws were placed and subtotal laminectomy of the target segment was performed. The local bone that was obtained during the subtotal laminectomy was finely trimmed and was filled with the interbody cage by the bone trimmer. The interbody cage with an autogenous bone graft was then inserted into the disc space for spinal interbody fusion. During the operation, surgical staff members were instructed not to exchange their gloves if there were no perforations. If glove perforation was noticed during the operation, the perforated glove was collected for data analyses; gloves worn in replacement of the initially perforated gloves were not collected for analysis. Upon completion of the surgical procedure, just before starting wound irrigation and closure, if no perforations were detected during the operation, outer gloves were removed and collected. After PLIFs completion, the inner gloves were removed and collected.

**Figure 1:**
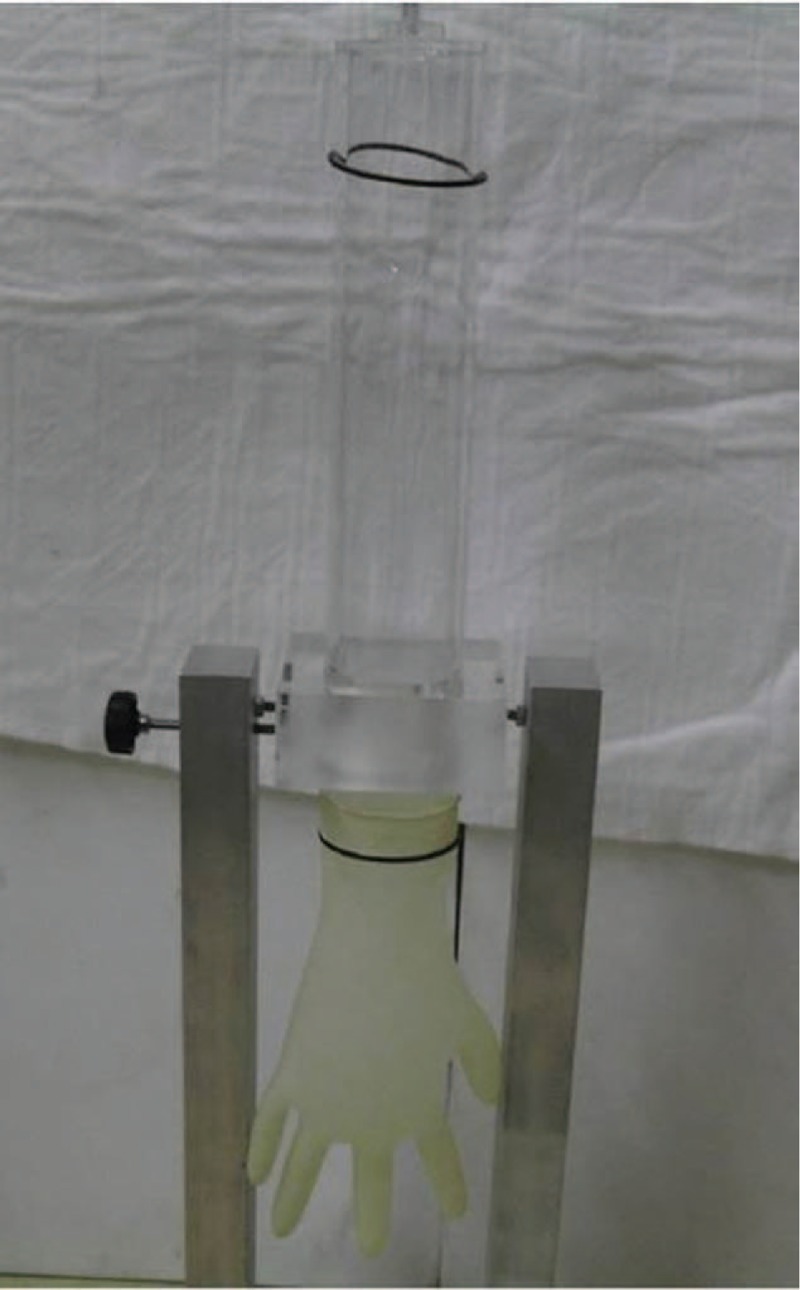
The Pinhole test (glove perforation test).

Collected gloves were subjected to the pinhole water infusion test after each operation (Fig. 1). The pinhole test based on the KSM 6640, was intended to find a perforation approximately 2 minutes after the infusion of 1000 ± 50 mL of water into the used glove.^[8]^ As a control, 37 new surgical gloves were similarly tested for leakage and found without perforation. Two blinded independent orthopedic surgeons observed the perforation rate and characteristics. The interobserver reliability of the glove perforation had the intraclass correlation coefficients (ICC) of 0.99 (95% CI, 0.98–0.99).

### 2.3 Statistical analysis

All statistical analyses were performed using SPSS Statistical Program, version 13.0 (SPSS, Inc, Chicago, IL). Chi-square tests were used to compare the glove perforation in relation to different baseline characteristics. The relative risk between the operative participants was also analyzed. Independent risk factors influencing surgical glove perforation were determined by multiple logistic regression analysis. The *P* values <0.05 were considered statistically significant.

## 3 Results

A total of 740 gloves were assessed in 37 PLIFs, the overall operative perforation rate which is percentage of detected more than one glove perforation event in all cases was 51.4% (19 out of 37 cases) and the overall glove perforation rate which is the percentage of perforated gloves in all gloves used for surgery was 3.8% (28 out of 740 gloves). Glove perforation was detected during the operation in 3.6% (1 out of 28 perforated gloves) and after the operation in 96.4% (27 out of 28 perforated gloves). The perforation rate was 1.9% for the inner gloves (7 out of 370 gloves) and 5.7% for the outer glove (21 out of 370 gloves) (Table 1). Only in one case, the outer and inner gloves were damaged at the same time on the left long finger (dorsal tip) of the bone trimmer.

**Table 1:**
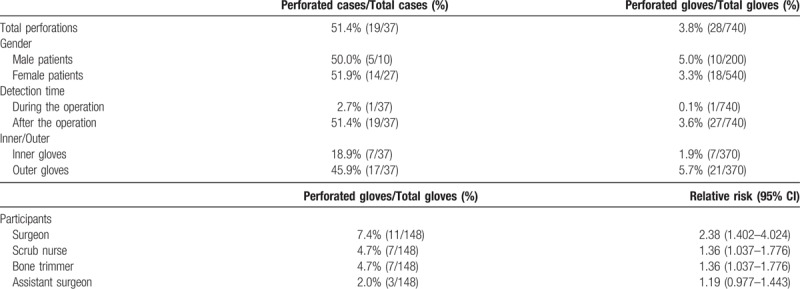
Summary of surgical glove perforation rate and relative risk in all operation staff.

The perforation rate of each surgical participant was 7.4% for the surgeon (11 out of 148 gloves), 4.7% for the bone trimmer (7 out of 148 gloves), 4.7% for the scrub nurse (7 out of 148 gloves), and 2.0% for assistant surgeons (3 out 148 gloves). In the surgical glove perforation of each surgical participant, statistical analysis showed that the relative risk was 2.38 for the surgeon (*P = *.002), 1.36 for the bone trimmer (*P = *.04), 1.36 for the scrub nurse (*P = *.04), and 1.19 for assistant surgeon (*P = *.13) (Table 1).

**Figure 2:**
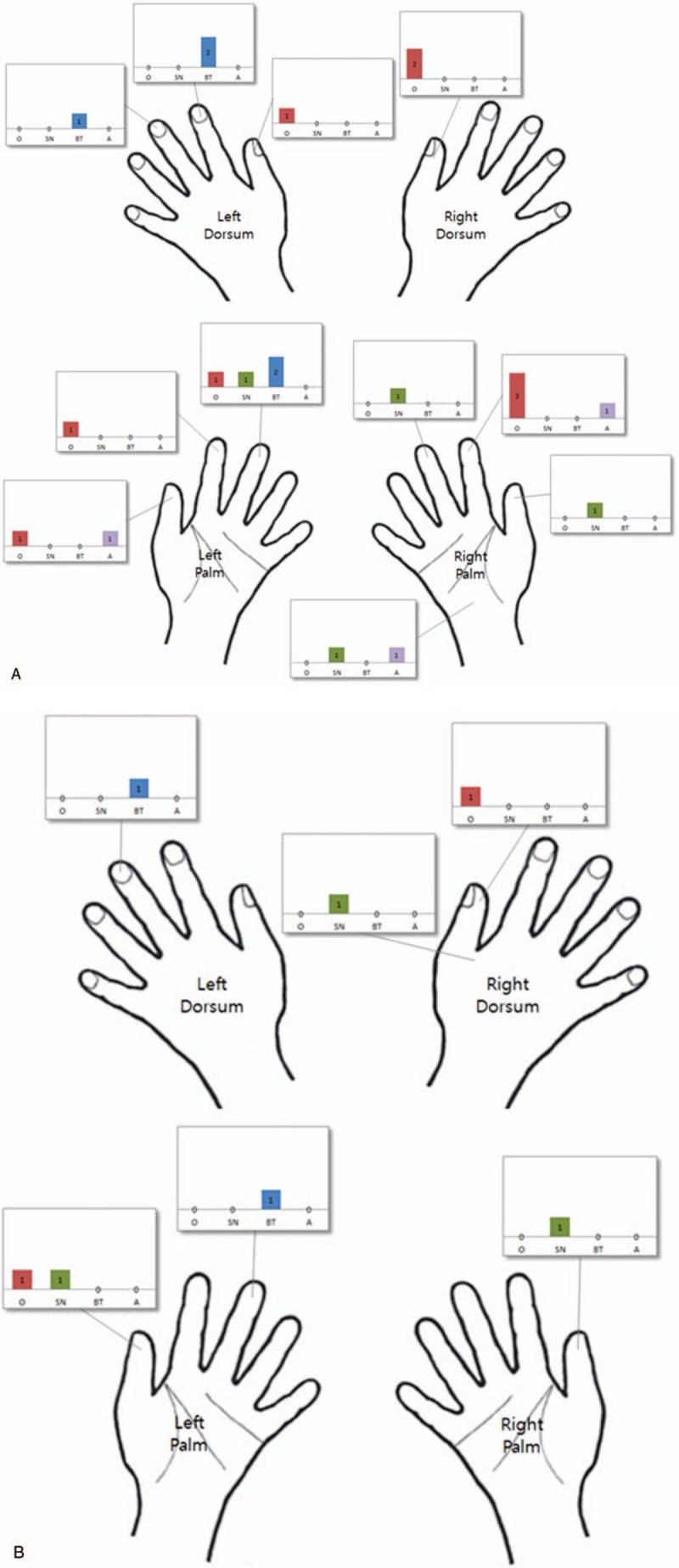
Schematic diagram showing the distribution of the perforation sites in outer glove (A) and inner glove (B) (O: surgeon, SN: scrub nurse, BT: bone trimmer, and A: assistant surgeon).

The most frequent perforation site in the surgeon's gloves was the volar side of the right index finger of the outer gloves (2%, 3 out of 148 gloves) and left thumb of the outer gloves (2%, 3 out of 148 gloves). Especially, only 1 case was detected glove perforation during the operation, there was occurred in the right index finger (volar side) in the surgeon's outer glove. Perforation of the bone trimmer's gloves tended to occur on the non-dominants hand, with most frequent perforations on the left long finger of the outer glove (2%, 3 out of 148) and the left long finger of the inner gloves (1.3%, 2 out of 148). In the scrub nurse, although the second showed a higher glove perforation rate, no most frequent perforation site could be identified. The detailed distribution of the outer and inner glove perforation sites according to participants was shown in Fig. 2A and B.

Among the 37 surgeries, the patients had a mean age of 67.3 ± 9.9 years (range: 51–85 years), mean body mass index of 24.8 ± 3.4 (range: 18.5–32.1). The mean fusion level was 2.0 ± 1.1 (range: 1–5, level-1: 16 cases/level-2: 11 cases/level-3: 6 cases/level-4: 3 cases/level-5: 1 case), mean operation running time was 144.2 ± 31.9 min (range: 110–200 min), mean estimated blood loss was 629.2 ± 477.5 cc (range: 100–2000 cc), and the mean volume of trimmed local bone was 7.3 ± 4.3 cc (range: 3–21 cc) (Table 2). The age, body mass index, fusion level, operation running time, and volume of estimated blood loss were not statistically significant in relation to surgical glove perforation. However, multivariate logistic regression analysis revealed that the volume of trimmed local bone is an independent risk factor for surgical glove perforation (ORs = 1.310, *P = *.02). Especially, glove perforation in the bone trimmer occurred especially frequently when the volume of trimmed local bone was greater than 9 ml. And, the more fusion level on the operating time, there was tended to increase the perforation frequency of scrub nurses gloves, although this effect did not reach statistical significance (ORs = 1.564, *P = *.18) (Table 2).

**Table 2:**
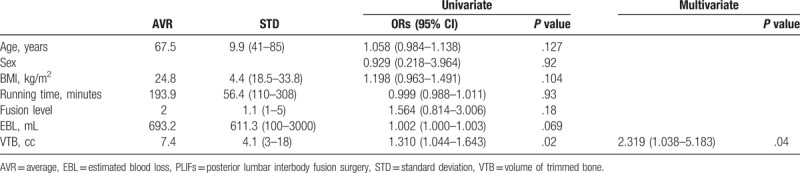
Independent risk factors for surgical glove perforation in PLIFs.

There was no evidence of blood-borne disease at all surgical staff member. Nineteen patients with the glove perforation event were also recovered without evidence of postoperative deep infection.

## 4 Discussion

This study investigated a characteristic of surgical glove perforation in the posterior lumbar interbody spinal fusion surgery. In 51.4% of all PLIFs (19 out of 37 PLIFs), glove perforations were found. Also, a glove perforation rate for 7.4% (11 out of 148 gloves) for the surgeon, 4.7% (7 out of 148 gloves) for the bone trimmer and scrub nurse respectively. Additionally, the perforation of surgical glove and the volume of trimmed local bone in this study demonstrated a significant correlation in the PLIFs. In the case was perforated surgical glove, the transmission of blood-borne disease and surgical site infection were not observed in both the surgical staff members and the patient.

Overall operative perforation rates were reported to vary from 8% to over 61% depending on the type of surgery.^[5,9]^ Orthopedic surgery, in particularly fracture, arthroplasty, and spinal fusion have a higher rate of perforation occurrence, possibly due to a greater likelihood of perforation from sharp bone fragment, the use of complex surgical instruments, and various bone structures.^[5,6,10–14]^ Since the introduction of the PLIFs by Cloward in the 1950s, the advent of durable interbody fusion cages and bone grafting alternatives has lessened the morbidity of bone graft harvesting and disc space collapse and has provided improved stiffness and stability to the affected spinal motion segment.^[15]^ Therefore, the procedure that is fine trimming of a local bone for use as interbody graft material is essential in the PLIFs. This study investigated the occurrence of perforation in outer and inner gloves used for PLIFs to determine if there was a difference between perforation rates according to the participants and which sites on the surgical gloves were most frequently perforated. Also, PLIFs can be considered as a surgical procedure in which surgical glove perforation is frequently encountered.

In the PLIFs, a 51.4% (19 out of 37 cases) overall operative perforation rate which is relatively higher than other surgical procedures was found. In a study by Ersozlu et al,^[3]^ a total number of 1528 gloves (622 inner and 966 outer) were examined, and overall glove perforation rate was 15.8% (242 out of 1528 gloves). As a conclusion, the authors recommended using routine double gloving during the orthopedic procedure to reduce the perforation of inner gloves. We also believe that wearing double gloving can reduce the risk of contamination through pre-existing perforations. Our study found that the overall glove perforation rate was 3.8% (28 out of 740 gloves) and outer gloves tended to be perforated more frequently than inner gloves (5.7% vs 1.9%). The perforation of the inner glove was observed as an isolated injury without the outer glove injury except for just one case. Only in one case, the outer and inner gloves were damaged at the same time on the left long finger (dorsal tip) of the bone trimmer. We found that postoperative detection of glove perforation was most common than intraoperative detection (96.4%, 27/28 perforated gloves versus 3.6%, 1/28 perforated gloves), as reported by Han et al.^[13]^ One case in which a glove perforation was detection during the operation is when the operator has damaged the glove with a surgical instrument in the process of subtotal laminectomy with momentary carelessness (Table 1).

**Figure 3:**
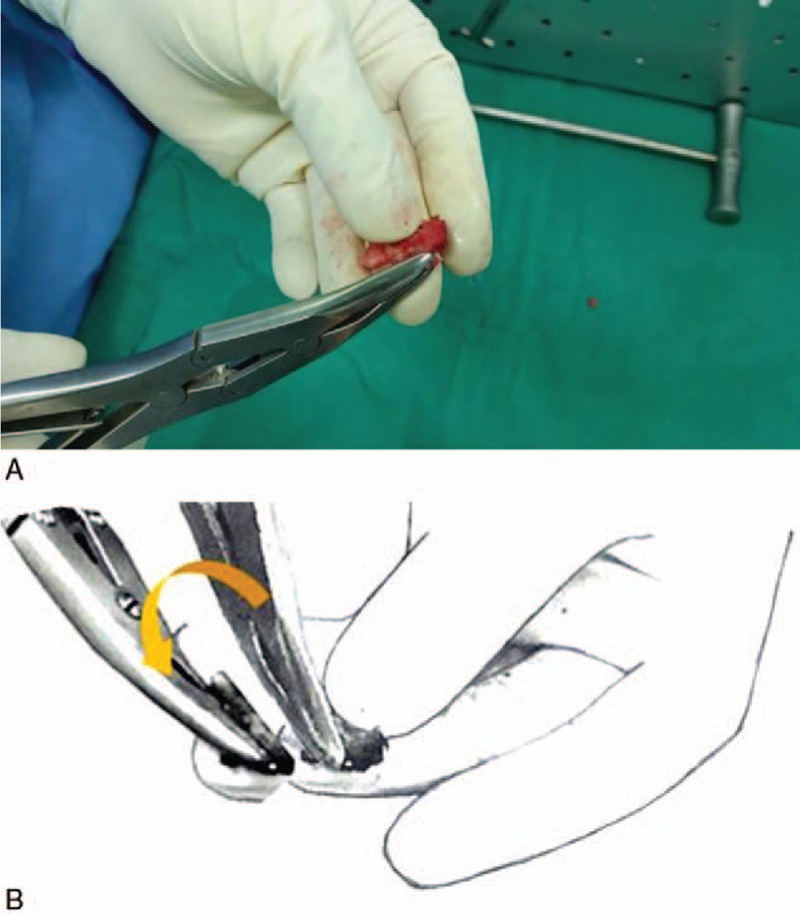
Intraoperative photograph (A) and schematic diagram (B) showing the bone trimming using the rongeur. The bone trimmer removed a soft tissue in an outward direction.

In this study, the relative risk of each participant and the contributing risk factors for surgical glove perforation were analyzed. Perforation occurred most often in gloves worn by the surgeon, followed by the scrub nurse and bone trimmer, which together accounted for 89.3% of perforation (25 of 28). The surgeon is the most important risk factor in glove perforation in PLIFs (relative risk: 2.38, *P = *.002). In the surgeon, there is a consensus in the literature that the non-dominant hand is more frequently affected than the dominant hand.^[16]^ We found was no difference between dominant (6 out of 11 perforated gloves) and non-dominants hands (5 out of 11 perforated gloves) of the surgeon. However, glove perforation of the surgeon was intensely observed thumb and index fingers, which are the highly used fingers (Fig. 2). In the scrub nurse, although the nondominant hand showed a higher glove perforation rate, common perforation site did not show a tendency. The only perforation sites in the bone trimmer's gloves were the long and index fingers of the non-dominant hand. The bone trimmer grasped the rough-hewn local bone fragment with the non-dominant hand and finely trimmed the local bone, removed soft tissue in an outward direction using a surgical instrument, such as a rongeur and knife with the dominant hand (Fig. 3A and B). While trimming local bone into the interbody cage, he fixes the mold for the interbody cage with the non-dominant hand.

Furthermore, the independent risk factors of glove perforation in PLIFs analyzed by logistic regression analysis. In previously published study, Patel et al^[17]^ reported that obesity is a prevalent condition in patients undergoing elective fusion for degenerative spinal conditions and may increase the prevalence and incidence of perioperative complications. However, we found no significant difference in glove perforation rate depending on age and body mass index. The overall glove perforation rate and operative running time in this study were significantly different from ordinary orthopedic surgery or spinal fusion surgery.^[3,4]^ As a result of this study, glove perforation was not correlated to the fusion level, volume of estimated blood loss, and operation running time. However, the volume of trimmed local bone was significantly correlated with glove perforation in the PLIF. Especially, glove perforation in the bone trimmer occurred especially frequently when the volume of trimmed local bone was greater than 9 ml (ORs = 1.310, *P = *.02). And, shorter running time per fusion level tended to increase the perforation rate of the scrub's gloves, possibly because of a loss of attention (ORs = 1.564, *P = *.18).

In the present study, we showed that heavily used fingers, which has area inevitably in contact with surgical instruments and sharp bones, are managed more easily. This result is important from the view point of safety for each patient and the surgical staff members. First, glove perforation is a definite theoretical pathway of blood-borne disease transmission. According to Palmar and Rickett,^[18]^ a surgeon risks more than one hepatitis infection per lifetime and more than 1 in 1500 surgeons is likely to be infected by HIV in the next 35 years because of damaged gloves. Second, glove perforation may lead to the development of osteomyelitis around the interbody space. In 2004, Carmouche and Molinari reported a case of infection around the interbody cage that occurred without wound infection in PLIFs.^[19]^ These authors presumed that this kind of infection occurs only through contamination of interbody spaces, grafted bone, or cages rather than through wound infection. Ahn et al^[20]^ reported that the incidence of surgical site infections was significantly higher in PLIFs than in posterior or posterolateral lumbar spinal fusion surgery. Given that 52% of infections that occurred in PLIFs lead to osteomyelitis around the interbody space and the local bone group had a higher infection rate, bacterial contamination of local bone chips and interbody space were presumed as additional causes that increased infections in PLIFs. In particular, perforation of the bone trimmer's gloves can lead to contamination of the interbody cage or grafts bone, which may progress to fatal complications. PLIFs may obtain a large amount of local bone through the removal of the spinous process and lamina than other types of spinal interbody fusion surgery such as transforaminal lumbar interbody fusion (TLIFs) and lateral lumbar interbody fusion (LLIFs). These modalities tend to use the allograft, demineralized bone matrix, or bone morphogenetic protein as interbody graft materials. For this reason, this study included only PLIFs. In some previous study reported that surgical glove perforations do not directly induce a postoperative infection.^[3,9,20]^ However, surgical gloving has been respected for efforts to reduce postoperative infection. Moreover, because of spine fusion procedure is performant surgery in lumbar degenerative disc diseases, we think that the efforts of systematic review and prevention of risk factors contributing the surgical site infections, are very important.

This study has some limitations. First, we performed a small volume data in a single center. To overcome this problem, we performed a prospective study and assessed by independent observers. Second, there might be a bias by using same glove products from the same company. Third, the follow-up periods were not long to confirm the long-term complications. Further investigations are needed with sufficient follow-up periods. However, the strengths of this study were that among the various spine surgeries, only PLIF surgery was included because of excluding possible bias from other spinal fusion surgery. Further, this study evaluated the bone trimmer's gloves manipulating the interbody cage or grafts bone, which may progress to fatal complications. To the best of our knowledge, there has been no published study in terms of the bone trimmer's glove.

## 5 Conclusion

PLIF is a surgical procedure in which surgical glove perforation is frequently encountered. The surgeon, scrub nurse, as well as bone trimmer are a higher risk factor for glove perforation and should always pay attention to glove condition. The heavily used fingers tend to show most glove perforation. Moreover, the volume of trimmed local bone was observed as an independent risk factor for glove perforation. Whereas the preparation of the interbody cage is essential for successful lumbar fusion surgery, the bone trimmer must pay attention to the glove perforation while performing this procedure.

## Author contributions

**Conceptualization:** Min Seok Kang, Jin Ho Hwang, Tae Hoon Kim.

**Data curation:** Yeong Ryung Lee, Eun Taek Jeong, In Seok Son, Tae Hoon Kim.

**Formal analysis:** Yeong Ryung Lee, Eun Taek Jeong.

**Investigation:** Min Seok Kang, Eun Taek Jeong, Tae Hoon Kim.

**Methodology:** Jin Ho Hwang, In Seok Son, Tae Hoon Kim.

**Supervision:** Suk Ha Lee, Tae Hoon Kim.

**Validation:** Jin Ho Hwang.

**Writing – original draft:** Min Seok Kang.

**Writing – review & editing:** Min Seok Kang, Jin Ho Hwang, In Seok Son, Suk Ha Lee, Tae Hoon Kim.
